# Patient-identified early clinical warning signs of nodular melanoma: a qualitative study

**DOI:** 10.1186/s12885-021-08072-4

**Published:** 2021-04-07

**Authors:** Adina Coroiu, Chelsea Moran, Jessica A. Davine, Kyla Brophy, Catherine Bergeron, Hensin Tsao, Annett Körner, Susan M. Swetter, Alan C. Geller

**Affiliations:** 1 Department of Social and Behavioral Sciences, Harvard T.H. Chan School of Public Health, 401 Park Drive, West Wing 4th floor, 403G, Boston, MA 02215 USA; 2 Department of Psychology, University of Calgary, Calgary, Canada; 3 Department of Educational and Counselling Psychology, McGill University, Montreal, Canada; 4 Harvard Medical School, Boston, USA; 5 Department of Dermatology, Massachusetts General Hospital, Boston, USA; 6 Department of Dermatology, Pigmented Lesion and Melanoma Program, Stanford University Medical Center, Stanford, USA; 7 Dermatology Service, Veterans Affairs Palo Alto Health Care System, Palo Alto, USA

**Keywords:** Nodular melanoma, Superficial spreading melanoma, Patient-identified early signs, Semi-structured interviews, Thematic analysis

## Abstract

**Background:**

Nodular (NM) and superficial spreading melanoma (SSM) show different disease trajectories, with more rapid development in NM and fewer opportunities for early detection often resulting in worse outcomes. Our study described the patient-identified early signs of thin NM via comparisons to *thin* (≤ 2 mm) SSM and *thick* (> 2 mm) NM.

**Methods:**

We conducted semi-structured interviews with NM and SSM patients and analyzed the data using thematic analysis.

**Results:**

We enrolled 34 NM and 32 SSM patients. Melanoma early signs uniquely identified by patients with thin NM included white, blue or black coloration, “dot-like” size, fast changes in shape and color observed over 2 weeks, elevation and texture or “puffiness” over 6–12 months, and the sensation that the mole “did not feel right”. Early signs reported by both thin NM and thin SSM patients included round or oblong shape, “jagged” border, pink/red, brown/reddish or dark coloration, “elevated like a pimple” or “tiny bump”, fast color darkening, diameter growth, and border irregularity, and mole feeling “really itchy”.

**Conclusions:**

We found evidence that early signs of NM can be self-identified, which has important implications for the earlier detection of this most aggressive type of melanoma by both health professionals and patients.

**Supplementary Information:**

The online version contains supplementary material available at 10.1186/s12885-021-08072-4.

## Introduction

Melanoma is the most common fatal type of skin cancer, and its incidence continues to rise [1, 2]. In the United States melanoma incidence increased from 20.7 per 100,000 in 2001 to 28.2 per 100,000 in 2015 [3]. Superficial spreading melanoma (SSM) and nodular melanoma (NM) are the most frequent subtypes, accounting for 80% of all diagnoses of cutaneous melanoma (CM) [4]. Newer histopathologic classifications of CM define these subtypes as occurring on skin without high cumulative sun damage (CSD), i.e., low-CSD melanoma, and SSM and NM are more likely to harbor the BRAF V600 mutation compared to other melanoma subtypes [5].

Tumour thickness at diagnosis is the key predictor of survival for CM, [6–8] and NM is usually thicker at diagnosis compared to SSM (median thickness at diagnosis: 2.19–2.6 mm for NM versus 0.54–0.6 mm for SSM) [9, 10]. While 90% of SSMs are diagnosed as thin tumours (<= 2 mm; T1/T2) only 20% of NMs are [10], with more than half (56%) of NMs diagnosed at a thicker stage (> 2 mm, T3/T4) [9]. Likewise, SSM accounts for 56% of invasive CM diagnoses and 30% of all deaths compared to NM, which accounts for only 14% of invasive diagnoses but 43% of all CM deaths [10]. Prognosis for thin NM is poorer compared to thin SSM [11], with reported rates for disease-free survival ranging from 82 to 84.9% for thin NM versus 91–96.4% for thin SSM [12, 13], although other studies show similar prognosis when matched for clinicopathologic factors.

Currently**,** it is unclear whether the increased thickness at diagnosis in NM can be attributed to sex- or age-based differences, e.g., NM tends to be diagnosed more often in males and in individuals 50 years of age and older [12, 14, 15]; potential delays in diagnosis due to atypical clinical presentation that does not fit general criteria for the early identification of problematic skin lesions [16, 17] e.g., the ABCDE rule -asymmetry, border irregularity, uneven color, large diameter (> 6 mm) and evolution [18–20] or the EFG rule -elevated, firm, and growing lesions [21, 22]; or whether NM is a biologically distinct, more aggressive subtype of melanoma that grows and spreads faster than other CM subtypes [4, 12, 23]. What is currently known is that NM tends to elude early clinical detection, with only a minority being detected early (T1/T2 stage) by dermatologists and most being identified later (T3/T4 stage) when melanoma may have already spread to regional lymph nodes [9]. Importantly, more NMs (44%) compared to SSMs (38%) are self-detected or first identified by family or friends as opposed to healthcare professionals [24, 25]. While physician-detected melanomas tend to be thinner compared to patient-detected lesions most melanoma are self-detected: by patients, spouses or friends [15, 26]. These data suggest a critical window of opportunity for early detection, in which patient perspectives can promote understanding of the early clinical signs of NM.

The few studies that have investigated early warning signs of NM versus SSM almost exclusively used quantitative methods either for data collection and/or data analysis (key findings summarized in the Additional file 1). As a result, detail about patient-recognized clinical features is limited to questions posed in descriptive, close-ended surveys or the availability of medical records data. Qualitative methods are patient-centered by design and are best suited to investigate patient perspectives on early signs and symptoms of medical conditions, such as melanoma, which develops with visible, pre-clinical signs [19]. Semi-structured interviews can explore patient narratives about early detection in greater depth, including key identifiable features, the circumstances that led to the identification of problematic lesions, and the patient’s knowledge base of the condition prior to detection. In addition, the use of prompts and guided questioning can improve patient recall [27].

### Research objective

To investigate more thoroughly the early signs of NM from the patient perspective, we conducted a qualitative study with semi-structured interviews focused on producing critical knowledge about mole appearance, observed changes in mole features over time, and sensations experienced about the problematic mole/lesion, as they became apparent to patients in the 12 months prior to diagnostic CM biopsy. This timeline for recall is common in melanoma prevention research and has been previously used to collect data on history of sunburns, sun exposure and practice of skin self-examination [28–32]. As per previous reports, a thickness cut-off of 2 mm was used to differentiate between thinner melanoma (≤ 2 mm, T1/T2) and thicker melanoma (> 2 mm, T3/T4). More specifically, we describe the patient-identified early signs of NM via comparisons to *thin* (≤ 2 mm) SSM and *thick* (> 2 mm) NM.

## Materials and methods

### Study design

The study employed a qualitative design with semi-structured interviews. Semi-structured interviews aim to explore individual viewpoints and the meaning behind people’s experiences to give a glimpse into the lived experiences as they occurred prior to theoretical explanations [33]. Individual interviews were conducted to collect patient-driven data addressing the main research questions. The goal was to develop a nuanced and comprehensive understanding of the clinical features of the problematic mole (or lesion) that participants identified on their own prior to receiving a diagnosis of melanoma. Study findings are reported as per the Standards for Reporting Qualitative Research (SRQR) [34]. The 21-item SRQR checklist is included as Supplementary Material.

### Participants and procedures

**Fig. 1:**
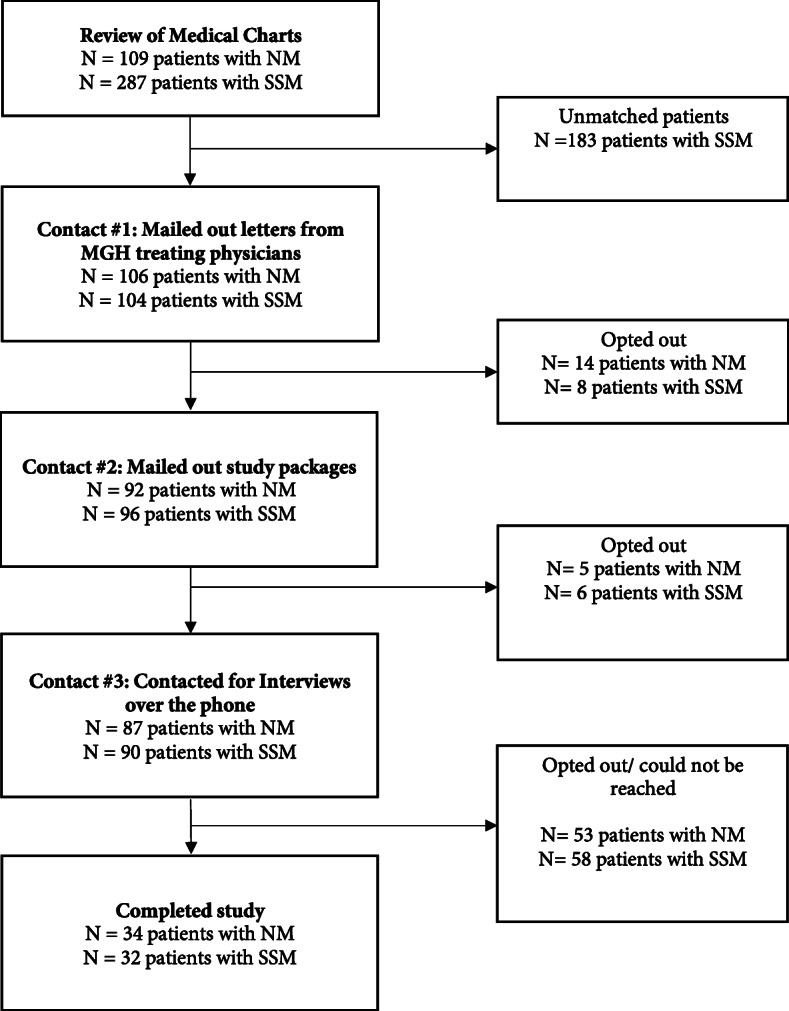
Study flowchart detailing study selection, enrollment, and completion. Legend. NM = nodular melanoma; SSM = superficial spreading melanoma; MGH = Masachussetts General Hospital

The study was approved by the Institutional Research Board (IRB) of Harvard T.H. Chan School of Public Health and the Research Ethics Board (REB) of McGill University, which are in agreement with the Declaration of Helsinki. Eligibility for the study included a confirmed diagnosis of either NM or SSM and receiving treatment at the Masachussets General Hospital between 2012 and 2017. Eligible participants were identified through medical hospital records and included men and women diagnosed with thin (≤ 2 mm) and thick (> 2 mm) NM and SSM. We identified all eligible NM patients (*n* = 109) and matched their profiles by sex, age at diagnosis, and melanoma thickness to SSM patients (1 NM to 3 SSM). The SSM matching pool was chosen randomly from a larger participant pool, as the MGH had disproportionate larger patient samples of SSM compared to NM, as is typical for these melanoma subtypes. Active enrollment occurred between December 2017 and April 2018. The flowchart of participation is included in Fig. 1.

Eligible participants received a letter via mail signed by their MGH treating physician informing them about the study and offering an opportunity to opt out of further communication about the study. Subsequently, eligible participants who had not opted out after the initial letter received a study package via mail, which included a brief study description, consent forms, and a brief demographic survey. The cover letter explicitly offered another chance to opt out of further study communication. Participants who had not opted out at this stage, were contacted via phone to discuss enrollment. All participants provided written informed consent prior to enrollment in the study. We planned to recruit approximately 80 patients (40 NM and 40 SSM) and continued scheduling interviews until we exhausted our sample of consenting participants. We reached out to patients five times before determining that they were inactive.

### Data sources

Data were collected via a brief sociodemographic survey and semi-structured interviews. The survey items inquired about key demographic characteristics (education level, age), and health behaviours and attitudes about melanoma prevention and early detection, and were administred solely to provide more context about the recruited sample and to help with contextualizing the qualitative findings. The interview guide (Additional file 1) included questions about the appearance of melanoma when initially spotted using prompts that were guided by the ABCDE criteria [18–20] which identifies problematic lesions by Asymmetry, irregular Borders, varying shades and Colors inside 1 mole, large Diameter (> 6 mm), and Evolution or changes in any of these criteria. We specifically asked about physical sensations experienced in or around the mole. The time of reference for reporting mole features and changes was 12 months prior to diagnosis. The same set of questions was posed to each participant, with prompts to facilitate recall, which allowed for flexibility to follow up on potentially relevant material. The interviews were conducted over the phone (CM), lasted between 20 and 40 min, were audio-recorded using open access software (Open Broadcaster Software), and were transcribed verbatim by a professional transcription service.

### Data analysis

Verbatim transcriptions of the interviews were imported into Dedoose [35], a type of software used for qualitative analyses. We used thematic analysis [36] and coded the interview data using an inductive-deductive method whereby a coding manual was created based on the review of the first 10 interviews (deductive approach), which was updated throughout the coding process, as new codes emerged (inductive approach). The initial round of coding was split among three coders (CB, CM, KB). The second round of coding was conducted by another coder (AC). Themes were generated and refined in an iterative fashion throughout multiple team meetings (AC, CM, JD, AG) held between September and December 2019. To ensure the trustworthiness and reproducibility of our findings [37], we used two criteria: *credibility* where the team reviewed the codes carefully in an iterative fashion and agreed on a final set of codes; and c*onfirmability*, where we challenged our own biases by practicing self-awareness throughout the coding process and by challenging personal assumptions during team discussions in which we collaboratively interpreted the data.

### Data synthesis

Data are presented in tabular format. Qualitative findings were summarized according to the established ABCDE criteria [18–20] and also incorporated discrete categories of data described by participants, including a) mole elevation (thickness or depth), b) perceived changes in any of the mole features and the chronology for observed changes; and c) clinical signs and symptoms experienced in or around the problematic mole. In line with the study aims, we used the thin NM group as our reference for contrasts with thin SSM and thick NM groups.

## Results

### Sample characteristics

The study sample comprised 66 patients: 34 patients diagnosed with NM (thin, *n* = 16; thick, *n* = 18) and 32 patients diagnosed with SSM (thin, *n* = 23; thick, *n* = 9) (see Table 1). Mean time elapsed from diagnosis to interview for the entire sample was 2.56 years (Mean _NM_ = 2.52; Median _NM_ = 2.26; Mean _SSM_ = 2.42, Median _SSM_ = 2.71). Patients with thin NM had the lowest mean age at diagnosis (56 versus 59, 61, 63). Across all four tumour thickness groups, most patients (> 50%) completed college. In addition, the majority of patients (*n* = 56; 85%) were diagnosed with their first melanoma; among patients diagnosed with a second melanoma (*n* = 10), 6 were NMs and 4 were SSMs. Self-reported rates for self-checking for melanoma in the 12 months prior to diagnosis ranged from 56% (thick SSM and thick NM) to 75% (thin NM). Self-reported rates for receiving a medical skin exam in the 12 months prior to diagnosis ranged from 22% (thick SSM) to 65% (thin SSM).

**Table 1:** Sample characteristics

Variable, % (n)	Nodular Melanoma		Superficial Spreading Melanoma	
	≤ 2 mm (*n* = 16)	> 2 mm (*n* = 18)	≤ 2 mm (*n* = 23)	> 2 mm (*n* = 9)
Sex, Female	62.5 (10)	22.2 (4)	52.2 (12)	22.2 (2)
Age at diagnosis, M (SD), Range	56.4 (14.3), 24–81	63.0 (11.4), 44–86	60.7 (17.7), 26–92	58.8 (7.5), 45–71
20–40	12.5 (2)	0.0 (0)	17.4 (4)	0.0 (0)
41–60	43.7 (7)	44.4 (8)	26.1 (6)	66.7 (6)
61–80	37.5 (6)	44.4 (8)	47.8 (11)	33.3 (3)
> 80	6.3 (1)	11.2 (2)	8.7 (2)	0.0 (0)
Highest education completed				
High school or GED	6.3 (1)	22.2 (4)	13.0 (3)	22.2 (2)
Vocational/ Technical	0.0 (0)	0.0 (0)	4.4 (1)	11.1 (1)
College graduate	37.5 (6)	27.8 (5)	39.1 (9)	44.4 (4)
Post-graduate or professional degree	56.3 (9)	50.0 (9)	43.5 (10)	22.2 (2)
Color of skin unexposed to the sun				
Reddish	6.3 (1)	22.2 (4)	9.1 (2)	22.2 (2)
Very pale	25.0 (4)	22.2 (4)	54.5 (12)	11.1 (1)
Pale with beige tint	62.5 (10)	44.4 (8)	31.8 (7)	33.3 (3)
Light brown	6.3 (1)	11.1 (2)	0.0 (0)	33.3 (3)
Dark brown	0.0 (0)	0.0 (0)	4.5 (1)	0.0 (0)
First melanoma				
Yes	75.0 (12)	88.9 (16)	82.6 (19)	100.0 (9)
Skin self-exam during the 12 months prior to diagnosis?				
No	25.0 (4)	44.4 (8)	34.8 (8)	44.4 (4)
Yes, whole body exam	25.0 (4)	16.7 (3)	13.0 (3)	11.1 (1)
Yes, partial exam	50.0 (8)	38.9 (7)	52.2 (12)	44.4 (4)
Medical skin exam during the 12 months prior to diagnosis?				
No	37.5 (6)	38.9 (7)	34.8 (8)	77.8 (7)
Yes, whole body exam	56.3 (9)	33.3 (6)	47.8 (11)	22.2 (2)
Yes, partial exam	6.3 (1)	27.8 (5)	17.4 (4)	0.0 (0)
Who performed the medical skin exam				
Dermatologist	37.5 (6)	38.9 (7)	47.8 (11)	22.2 (2)
PCP or another HCP	25.0 (4)	27.8 (5)	17.4 (4)	0.0 (0)

*PCP* primary care provider, *HCP* health care provider

Patients with thin NM reported fewer physical symptoms experienced in the 12 months prior to diagnosis, such as itching, bleeding, irritation, or pain compared to patients with thin and thick SSM or thick NM. Further, there were no reports of tenderness of the mole, discharge or peeling among thin NMs. Half of patients with thin NM self-discovered their melanoma compared to approximately 40% of thin SSM and > 75% of thick NM and thick SSM. Approximately half of patients with thin NM reported some confidence (“somewhat”, “quite”, or “extremely” confident) in identifying problematic moles compared to 1/3 of patients with thick NMs and thick SSM and ¼ of thin SSM’s. More than 3/4 patients from each group self-identified as “generally paying attention to their health”. Descriptive statistics are included in Table 2.

**Table 2:** Self-report survey variables crosstabulated by Melanoma type and depth

Variable, % (n)	Nodular Melanoma		Superficial Spreading Melanoma	
	≤ 2 mm (*n* = 16)	> 2 mm (*n* = 18)	≤ 2 mm (*n* = 23)	> 2 mm (*n* = 9)
Physical signs in 12 months pre- diagnosis (*checked all that apply*^a^)				
Itching	12.5 (2)	22.2 (4)	17.4 (4)	44.4 (4)
Bleeding	12.5 (2)	22.2 (4)	17.4 (4)	44.4 (4)
Irritation^b^	12.5 (2)	33.3 (6)	4.3 (1)	66.7 (6)
Tenderness^b^	0.0 (0)	38.9 (7)	4.3 (1)	55.6 (5)
Pain^b^	6.3 (1)	33.3 (6)	4.3 (1)	55.6 (5)
Discharge	0.0 (0)	5.6 (1)	4.3 (1)	11.1 (1)
Peeling	0.0 (0)	33.3 (6)	4.3 (1)	11.1 (1)
Who discovered the melanoma?				
Self or partner	50.0 (8)	76.5 (13)	39.1 (9)	87.5 (7)
Friend or colleague	0.0 (0)	11.8 (2)	8.7 (2)	0.0 (0)
Primary care physician	12.5 (2)	0.0 (0)	13.0 (3)	0.0 (0)
Nurse or physician assistant	0.0 (0)	0.0 (0)	4.3 (1)	0.0 (0)
Dermatologist	31.3 (5)	5.9 (1)	26.1 (6)	0.0 (0)
Another professional	6.3 (1)	5.9 (1)	8.7 (2)	12.5 (1)
Confidence differentiating healthy and problematic moles?				
Not at all confident	25 (4)	44.4 (8)	17.4 (4)	33.3 (3)
A little confident	18.8 (3)	22.2 (4)	47.8 (11)	33.3 (3)
Somewhat confident	18.8 (3)	11.1 (2)	13.0 (3)	22.2 (2)
Quite confident	31.3 (5)	16.7 (3)	13.0 (3)	11.1 (1)
Extremely confident	6.3 (1)	5.6 (1)	8.7 (2)	0.0 (0)
“I am someone who pays attention to my health”				
Disagree	0.0 (0)	5.6 (1)	0.0 (0)	22.2 (2)
Neither agree or disagree	12.5 (2)	22.2 (4)	17.4 (4)	0.0 (0)
Agree	31.3 (5)	50.0 (9)	39.1 (9)	33.3 (3)
Strongly agree	56.3 (9)	22.2 (4)	43.5 (10)	44.4 (4)

^a^The percentages do not add to 100% within each thickness group because the respondents were instructed to select all of the symptoms they had experienced from a list of possible symptoms

^b^We found statistically significant differences between the four thickness groups

A summary of qualitative findings pertaining to the self-identified early signs of melanoma is included in Table 3. For brevity purposes, the thick SSM group (*n* = 9) was not included in the qualitative analysis, as it offered no new information beyond what was already provided by the other two comparison groups, thin SSM and thick NM.

**Table 3:** Perceived early signs and symptoms of Melanoma

Signs/ symptoms	Nodular melanoma	Superficial spreading melanoma	Nodular melanoma
	≤ 2 mm	≤ 2 mm	> 2 mm
**Asymmetry**			
Round	Round or roundish, circle or circular	Circular, like a big circle	Circular, like a big circle
Oblong	Not perfectly round, oblong	Not perfectly round, jetted off	
	Like a small kidney bean	Not perfectly round, smaller in one direction	
Square	Square		Rectangular
**Border**			
Slightly irregular	A little bit (or slightly) irregular	Half-moon edge on the side of the border	A little irregular
			Definite border, visible where it started and stopped
Jagged	Jagged	Jagged border on one side	
		Jagged, uneven, undefined, melted into skin	
Irregular coloration		Skin was a little bit pink, right on the border	
		[Pinkish with] a tan border	
**Color**			
White	Little white dot		Interior looked white or grey-white
			Brownish white
			Darker white
Beige	Very light beige w/ black spot in the middle		Skin color shade
Blue	Bluish dark		
	Bluish, multi-colored		
Black	Black freckle, like a black head		
	Black dotted marks leaving a trail of brown		
Pink	Pinkish, looking like a pimple	Pinkish	Pink
		Pinkish with a tan border	
		Pinkish, pearlescent- like a reflection of a pearl	
		Pink bumpy/bubbly area with a black freckle on top	
			Dark pink
Red	Red		Red
	Red, pinkish lesion	Reddish	Reddish, almost bright red
	Tiny, little red spot		
Brown		Light and dark brown, with darker spots inside	
	Brown	Brown	Brown
	Brownish or maroonish	Brownish like a dark freckle	A little brownish
	Brownish dark		Dark brown
Reddish brown	Dark, reddish brown	Brown and lighter /reddish	Brown with some red
		Reddish brown	Brown with some purple
Dark	Really dark with an even darker spot inside	Dark, almost black	Dark
**Diameter**			
Tiny dot	Like a dot made with a pen	Tiny, like lead on a pencil, the size of a dot	
	Tiny, tiny little spots		
	Tiny, tiny, tiny, like the head of a pin		
	Like if you took a fine-tipped pen and you just put three dots on a piece of paper		
< Pencil eraser	Fairly small (1/8 of an inch)	Very small (2 mm to 3–4 mm)	Like a small pinhead used for sewing (1.5 mm)
	Much smaller than a pencil eraser	Much smaller than a pencil eraser	Like the tip of a pen (2 mm)
	Like a half of a pencil eraser	Smaller than the size of a pencil eraser	
	About 2/3 of a pencil eraser	Half the size of a pencil eraser, very small	
~ Pencil eraser	The size of a pencil eraser	Almost the size of a pencil eraser	The size of a pencil eraser
		The size of a pencil eraser or a little smaller	Like a big pimple (1/4 of an inch
			About 1/4 in. round
> Pencil eraser	A little bigger than a pencil eraser	Like two pencil-head erasers side-by-side	A little bigger than the size of a pencil eraser
	The size of my little finger’s fingernail	The size of the little fingernail on your hand	As big as a very small blueberry, maybe even smaller
	Really tiny, smaller than 1 cm	1 cm diameter	
		Smaller than the size of a dime	
		A little bit smaller than my thumbnail	The size of my thumbnail
		About the size of a dime, maybe bigger	About the size of a dime
			The size of a penny
			The size of a quarter, large
			A little bigger than a quarter
			Approximately 2 cm
**Elevation**			
Slight	Teeny little bump	A little raised (1/8 of an inch; 1 mm)	A little raised
	Only slight [elevation]	Elevated a little, you could feel it, definitely	A little raised bump
	Not huge, just slight [elevation]	Could feel it- if you ran your finger over it	A little bit raised but not grossly
	Definitely more flat, but [also] raised a bit	Not very much elevated, a little bumpy	Elevated a little bit
	Flat, less than 1 mm, a really tiny thing	Like a little raised scar, bubbly a little bit	A little bit elevated, some parts higher than others
	A little bit elevated but small, small	Rounded at the top, a tiny bubble like a tiny curve -also went down below the surface	Could feel it, wasn’t flush with the skin (1/4 in. high)
	Elevated above the skin like bumps on skin		The big the balloon was maybe 1/8 of an inch
	Elevated, like a pimple		Raised at least a ¼ of an inch, maybe more
Textured	A little bit raised, puffy, just like a little bump	A little bit bumpy with a rough texture	Growing out of the skin, I could feel the crustiness
	A little bit raised, puffed up	A little raised, a little crusty	Felt like a bee sting, no pain (the texture of it)
Prominent	Raised up and prominent		Raised, pronounced, thick (5 mm)
			Raised, like a swelling from a bee sting
**Evolution** ***[chronology]***			
Asymmetry change	From round to oblong *[2 weeks]*	Changed shape *[In a matter of weeks]*	Looked different from last time I checked *[2 months]*
		Not the same shape as in the past *[6 months]*	
Border change	Became more irregular *[6–12 months]*	Got irregular *[In a matter of weeks]*	
		Some parts of the border became red *[Over the last few years]*	
Color change	From light beige to beige with a black spot *[Over time]*	Became darker at the center	
	From brownish to darker with brown tinges *[2 weeks]*	From light brown to really dark *[Really fast]*	
		Got darker, from light to dark brown *[6 months]*	
	Became a little bit dark	Got a little darker *[Slowly, over the years]*	
			From brown to brown with purple in it *[2–4 months]*
	From brown to black, in a dripping pattern *[2 weeks]*		From brown to black *[4–5 months]*
	From bluish dark to almost black *[3 months]*		Blackened
Diameter change	Didn’t get too much bigger *[2 weeks]*	Grew in size *[Overnight]*	Got bigger, from 0.5 to 2 cm *[4 weeks]*
	Grew quickly, all of a sudden *[4–6 weeks]*	Got a little bigger *[Over the last few years]*	Got bigger *[3–4 months]*
	Got a little bit bigger, larger *[3 months]*	Grew a little bit bigger *[Slowly, over the years]*	Came back/grew after biopsy *[4–5 months]*
	Got bigger, from 1 to 2 mm *[6–12 months]*	Grew in size, from nothing to pencil eraser size *[In a matter of weeks]*	Got slightly bigger *[Almost 1 year]*
	Got (a little bit) bigger *[Over time]*		Kept getting bigger and bigger
	Growing in size *[12 months]*		
Elevation change	Got raised *[2 weeks]*		Got higher *[4 weeks]*
			More raised *[2 months]*
		Got more density to it *[Very quick]*	Became thicker *[3.5 weeks]*
	Became more pronounced		Became more pronounced, protruding from the skin *[Relatively quickly]*
	Became puffy *[6–12 months]*		Became bumpier, not smooth *[2–4 months]*
	Puffed up *[Over time]*		
**Physical signs and symptoms**			
Itchy	A little bit itchy		
	Really itchy	Itching a good deal	
	Became itchy	Became itchy *[6 months]*	Became itchy *[2 months]*
Bleeding	Bleeding after shaving/ picking at it		Bleeding after shaving or squeezing *[2–3 weeks]*
	Blood spots under the mole		Bleeding
Weeping			Weeping pus
			Discharge
			Moist
Multiple signs	A little bit itchy and bleeding *[Once]*	Itchy, scaling and flaky, cracking, bleeding	Itchy, sore/sensitive, and bleeding- from towel drying
			Bleeding a little, open sore, scabbed over
		A little bit itchy and a little bit scaly	Itchy and erupting *[Periodically]*
		Itchy, did not heal, looked like a keloid scar	
		Became dry, scaly, peeling *[All of the sudden]*	
		Sensitive and hurting/ sore, radiating pain *[All of a sudden]*	Itchy and painful
			Oozing- from towel drying, sensitive, breaking open
Tactile sensations	Did not feel right, it was purely tactile		Could feel it- by touching
		Hardened, became more solid *[Over a few days]*	Hardened a little bit
			Felt like a hard pimple- by touching
			Felt like cracking a peanut open- after squeezing

*NM* nodular melanoma, *SSM* superficial spreading melanoma

### Self-identified early signs of melanoma that are unique to nodular melanoma

With respect to mole appearance, thin NM’s stood out in terms of coloration, e.g., “white”, “blueish dark”,” blueish, multi-colored”, or “black”, and diameter, e.g., “tiny, tiny, little spot” or “little white dot”. In addition, thin NM’s reported fast changes in shape, e.g., from “round” to “oblong”; fast changes in color, e.g., from” brownish” to “darker with brown tinges” or from “brown” to “black, in a dripping pattern”; and developed vertical growth over the period of 2 weeks. Other changes unique to thin NM, which reportedly occurred over the course of several months to 1 year, included changes in color, e.g., from “blueish dark” to “almost black”, and the development of texture, e.g., “became puffy”, “puffed up”.

Thin NM patients reported elusive tactile sensations, such as “did not feel right, it was purely tactile” compared to more defined signs reported by thin SSM: “hardened, became more solid”, and by thick NM: “felt like a hard pimple” or “felt like cracking a peanut open.” Bleeding was characteristic of both thin NM and thick NM, with “blood spots under the mole” reported solely by thin NM while “bleeding after shaving or picking at the mole” was reported by both groups. Bleeding was not reported among the thin SSM group.

### Self-identified early signs of both nodular and superficial spreading melanoma

Thin NM and thin SSM reported both symmetric e.g., “round“, “circular”, and asymmetric shape, e.g., “oblong”, “like a kidney bean”, “not perfectly round, jetted off”; border irregularity, e.g., “a little bit irregular”, “jagged”; coloration in the pink-red-brown range, e.g., “pinkish”, “reddish”, “brownish”, “reddish brown”, “dark”; diameter ranging from “much smaller than a pencil eraser” to “[ …] the size of the little fingernail”; and small elevation, e.g., “tiny little bump”, “elevated like a pimple”. Both thin NM and thin SSM reported fast changes in diameter occurring over a few weeks period, and changes observed over the course of six to 12 months in border irregularity, e.g., “got irregular”, color darkening, e.g., from lighter to darker shades of brown, and the development of itchiness, e.g., “itchy”, “really itchy”.

## Discussion

Early detection of the more rapidly-growing NM subtype is critical to improved patient outcomes. By the time a patient’s NM shows ABCDE criteria, it is likely to be thicker at diagnosis and less curable. We employed qualitative methodology to facilitate recall of the patient-identified clinical features of problematic moles observed in advance of a formal melanoma diagnosis. This work has important implications for the early detection of NM, which was previously thought to be undetectable at earlier stages.

Our study included 66 patients with NM and SSM, which is the largest and only second [38] qualitative study to date with this population. This study found several patient-identified early signs of melanoma that were unique to thin NM (≤ 2 mm), including small white dot, visible blood spots underneath the mole, blue mole darkening fast, round mole becoming asymmetric fast, mole developing elevation fast, mole becoming puffy and crusty over time, and an overall physical sensation that the mole is different from other moles. Common criteria used for the early identification of melanoma, such as the ABCDE [18–20], elevated-firm-growing (EFG) [21, 22] or the blue-black (BB) rule [39] capture some of the early features of NM identified in our study; however, white coloration and very small diameter are not adequately represented in any of these mnemonics.

Additionally, this study found some overlap between the patient-identified early signs of NM and SSM, including round and asymmetric shape, red or brown mole, raised pink bump, darkening of the mole, border becoming irregular, and itchiness developed over time. Symmetric round shape, small diameter (< 6 mm), and itchiness are not captured in the ABCDE criteria; however, elevation or vertical growth are included in the EFG mnemonic, which is typically used to identify NM and less commonly used for the early identification of SSM. A 2003 brief by Kelly and colleagues [21] noted higher percentage of symmetric nodular melanomas (90%) and regular borders and single coloration (78%)- compared to superficial spreading melanoma- and the appearance of a round nodule growing vertically from the onset.

While smaller size diameter and changes in shape, border, color, diameter, elevation and itchiness have been previously reported as features of NM [38, 40–42], this is the first study to provide patient-reported chronology for observed changes in mole features. Specifically, among early NMs, changes in mole shape, darkening of color, and rapid vertical growth reportedly occurred over a 2 week period, accompanied by tactile sensations suggestive of “something different and potentially problematic” about the mole.

### Limitations

Given this study asked retrospectively about the early signs of melanoma, there may be a concern about the accuracy of patient recall given the interval between the onset of signs/symptoms and the patient interviews. Prior results from a large nested case-control study investigating the impact of recall bias on effect estimates for various self-reported melanoma risk factors suggested some evidence of bias, with the overall conclusion that the length of time between diagnosis and interview did not systematically affect recall [43]. In a qualitative study asking about retrospective memories, it is virtually impossible to gauge the impact of recall bias. In our study, time from diagnosis to the interview did not differ substantially across the three groups included in the qualitative analysis, thin NM, thin SSM, thick NM, which suggests the accuracy of self-reported data might be comparable across the groups. Notably, results from our written survey show that 5 patients with thin NM (5/16, 31%) reported clinical signs and symptoms. Findings from interview data show that 8 patients with thin NM (50%) reported clinical signs and symptoms: bleeding alone (*n* = 3), itching alone (*n* = 2), itching and bleeding (*n* = 2), and an undefined tactile sensation accompanied by the appraisal that “did not feel right” about the mole (*n* = 1). The discrepancy between the two data sources could be explained by extensive prompts employed by the interviewer to facilitate recall and speak to the relevancy of our qualitative methodology to provide meaningful and personalized information. Last, patients’ awareness of individual risk factors (e.g., personal or family history; phenotypic features) could affect people’s perceptions of the disease, including readiness to examine the skin for the early signs of skin cancer. In this study, we did not examine patients’ knowledge of risk factors.

### Future directions for research

Results from our formative study can guide the development of quantitative measures to assess early detection of nodular and superficial spreading melanoma, which would allow for further quantification of rates of self-identified early features of melanoma. Our results could also guide future research to develop educational materials about the early detection of various types of melanoma, including the NM subtype, which appears to be more amenable to earlier detection by patients than previously claimed. Further validation of our findings may then warrant revision of existing criteria for earlier clinical recognition of the NM subtype.

## Conclusions

Overall, our findings indicate that some of the patient-identified early signs of thin nodular melanoma are not currently ascribed to any of the existing mnemonics used for the early identification of melanoma (ABCDE, EFG, BB rule). These specific features from our findings include the appearance of a small persistent bump or pink pimple, or a tiny round nodule of white, blue, or black color, which feels itchy and undergoes rapid changes in appearance, and “feels” noticeable over a brief 2 weeks. Incorporation of These findings could inform future development of educational materials on the early detection of melanoma, especially as it pertains to the key warning signs of nodular melanoma. Nodular melanoma is a less common but more fatal melanoma subtype, that has typically eluded early detection strategies and occurs more frequently in older white men [44, 45] and across various racial-ethnic groups, such as Hispanic whites [9, 46]. Individuals at high risk as well as healthcare professionals involved in their care particularly benefit from learning about these early signs of nodular melanoma amenable to self-identification.

## Supplementary Information

**Additional file 1: Appendix A: Table S1.** Clinical Features of Nodular Melanoma (NM) and Superficial Spreading Melanoma (SSM), as per Previously Published Reports. **Appendix B.** Interview Guide.

**Additional file 2.** Standards for Reporting Qualitative Research (SRQR) Checklist.

## Data Availability

The datasets used and/or analysed during the current study available from the corresponding author on reasonable request.
