# Supplementary material for: Patient-identified early clinical warning signs of nodular melanoma: a qualitative study
Source: BMC Cancer. 2021 Apr 7;21:371. doi: 10.1186/s12885-021-08072-4 (PMC8028760; doi:10.1186/s12885-021-08072-4)
Supplement: Supplementary file 1 — Additional file 1: Appendix A: Table S1. Clinical Features of Nodular Melanoma (NM) and Superficial Spreading Melanoma (SSM), as per Previously Published Reports. Appendix B. Interview Guide. [file 12885_2021_8072_MOESM1_ESM.docx]

Supplementary Materials:

Appendix A

Table S1. Clinical Features of Nodular Melanoma (NM) and Superficial Spreading Melanoma (SSM), as per Previously Published Reports

| Author, year, country | Study Design | Population | Sample size | Group Comparisons | Main Findings |
| --- | --- | --- | --- | --- | --- |
| Chamberlain, 2003 ^36^  Australia | Cross-sectional | NM, SSM | NM, n=33  - *n=18, < 3 mm*  *- n=15, >= 3mm*  SSM, n=92 | NM vs SSM  NM < 3 mm vs.  NM >= 3 mm | - NM less likely to be pigmented, to report change in color  - NM more likely to be symmetric, to show 1 uniform color, red or pink, elevated, weeping/bleeding, crusting/ “catching on clothing”, tenderness  - Similarities: shininess, opaqueness, or crustiness; change in size/shape/color, change duration, crustiness, pain, itching  - NM < 3 mm more likely to have smaller diameter, pink or red color  - NM >= 3 mm more likely to show higher diameter, history of change in size, shape |
| Bergenmar, 2002 ^32^  Sweden | Semi-structured interviews | NM, SSM | NM, n=22 (≤ 2 mm)  SSM, n=32 (≤ 2 mm) | NM ≤ 2 mm vs.  SSM ≤ 2 mm | - NM smaller diameter, more often as new lesion, discharge  - Similarities: mole looking different; itching and bleeding; uniform color; changes in border (“becoming more diffuse”, “wavy contour”, “ulcerated border”), surface/texture (“rough”, “dry”, “blistered”, “partly smooth”), color, diameter, and elevation |
| Geller, 2009 ^38^  Australia | Cross-sectional | NM | NM, n=369  - n=209, ≤ 2 mm  - n=160, > 2 mm | NM ≤ 2 mm vs.  NM > 2 mm | - NM <= 2 mm more likely to have an irregular shape, brown pigmentation, and report change in color  - NM > 2 mm more likely to be lumpy/raised or looking like a blister, red/pink or, pale/no pigmentation, and bleeding or weeping  - Similarities: change in size, irritation, onset of itchiness, the lesion being ‘‘different from other spots’’ or having ‘‘just appeared’’ |
| Kalkhoran, 2010^35^ International sample | Case series | NM | N=11 (<1.3 mm) | None | - A: mostly symmetric (7/11)  - B: some regular, some irregular  - C: black, brown-black, brown, grey-brown, blue, red, pink  - D: small (range 3-10 mm)  - e: all elevated  - Shape: round, oval, irregular |
| Bono, 2011 ^34^  Italy | Retrospective case review  (Data sources: medical records) | NM | N=11  (Diameter < 6 mm) | None | - A: mostly symmetric  - B: well-defined border, regular or irregular  - C: black/ black-red/ black-tan; brown-tan; blue-red; red; pink/ pink-tan  - D: small (range, 2-6 mm)  - e: all elevated (range, 0.5-2.4 mm)  - Change in size (n=2) |

*Note*. A = asymmetry, B = border irregularity, C = color variegation, D = diameter; e = elevation.

Supplementary Materials:

Appendix B: Interview Guide

**What can you recall about the problematic mole when you first spotted it? [Prompts]:**

*[RE: Salient feature]*

- What “bothered” you about this mark the most?
- Was there something that stood out for you, about this mole?

*[RE: Means of detection-ugly duckling, ABCDE]*

- Did this mole look different than your other moles?
- Was the mole new or you’ve had it for a while?
- What rules did you use in detecting abnormal moles?
  - Ugly duckling – ever heard of that before?
  - ABCDE criteria – how about this?
  - Which one helped you to spot this (or other) lesion?

**What can you tell me about how this skin mark looked like when you first spotted it?**

*[RE: Elevation]*

- Can you tell me something about the shape of this mole (skin mark)?
- Can you remember if it was flat and spread out over the skin, or was it elevated above the skin?
- Were some parts higher than others?

*[RE: Asymmetry]*

- Was it round? Flat? Uneven shape? Looked similar/different on both sides?

*[RE: Border]*

- What did the border look like? (i.e. crusty or notched)
- Was there anything special about the border or edge of the mole?

*[RE: Color]* What can you remember about the color?

- What color was it? Or non-pigmented? (i.e. red or pink)
- Was the whole mole the same color, or did the mole have different colors?

*[RE: Diameter]* What was the size of the mole?

- What diameter/size was it? *Use the “pencil eraser” analogy.*

*[RE: Symptoms / sensations]*

- Do you recall experiencing any sensations like itching? Scaling? Bleeding? Other symptoms?
- Other symptoms include: Bleeding, Irritation, Tenderness, Pain, Discharge, Peeling, Tenderness
- Is there anything else that you can recall that stood out for you, in terms of how the spot looked like? or how it felt like?

***[RE: Evolution/Changes]* What kind of changes did you (or someone else) notice over time, if any?**

- How long ago did you start noticing changes in the mole?
- Did it become more noticeable or different than other skin signs?
- How did it look like over time, what exactly changed?
  - Size?
  - Color?
  - Shape?
  - Depth?
- Sensations (i.e. begging to scab or bleed)
  - - Itching?
    - Peeling?
    - Bleeding?
    - Discharge?
    - Irritation?
    - Tenderness?
    - Pain?
